# Supplementary material for: Rhopalocnemis phalloides has one of the most reduced and mutated plastid genomes known
Source: PeerJ. 2019 Sep 12;7:e7500. doi: 10.7717/peerj.7500 (PMC6745192; doi:10.7717/peerj.7500)
Supplement: Supplemental Information 1 [file peerj-07-7500-s001.pdf]

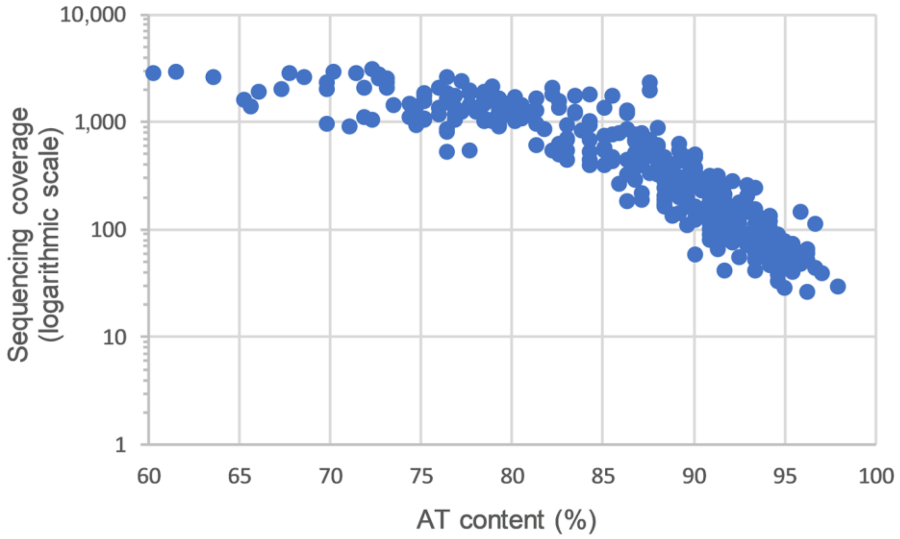

Figure S1. Dependence of the sequencing coverage on the AT content in the plastid genome of *Rhopalocnemis phalloides*. The sequencing coverage is provided on a logarithmic scale. The window size for the AT content calculation was 241 bp (the average insert size). The step for window sampling was 50 bp.

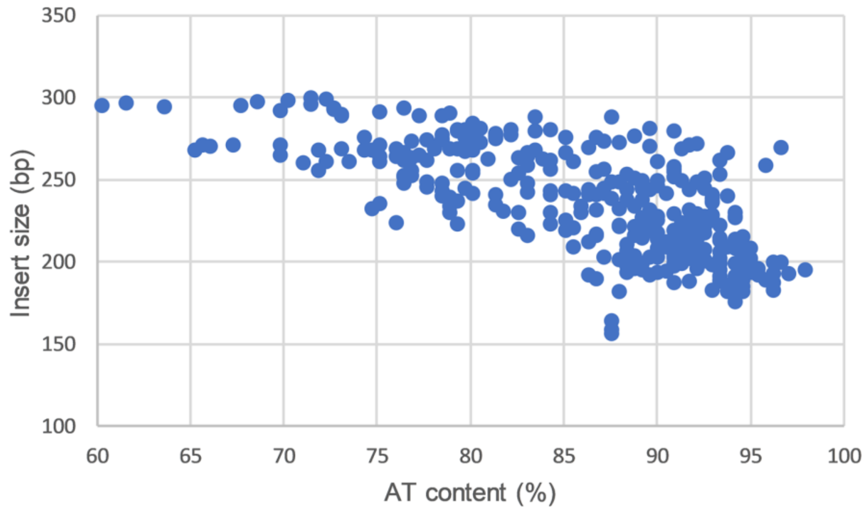

Figure S2. Dependence of the insert size on the AT content in the plastid genome of *Rhopalocnemis phalloides*. The window size for the AT content calculation was 241 bp (the average insert size over the whole genome). The step for window sampling was 50 bp.

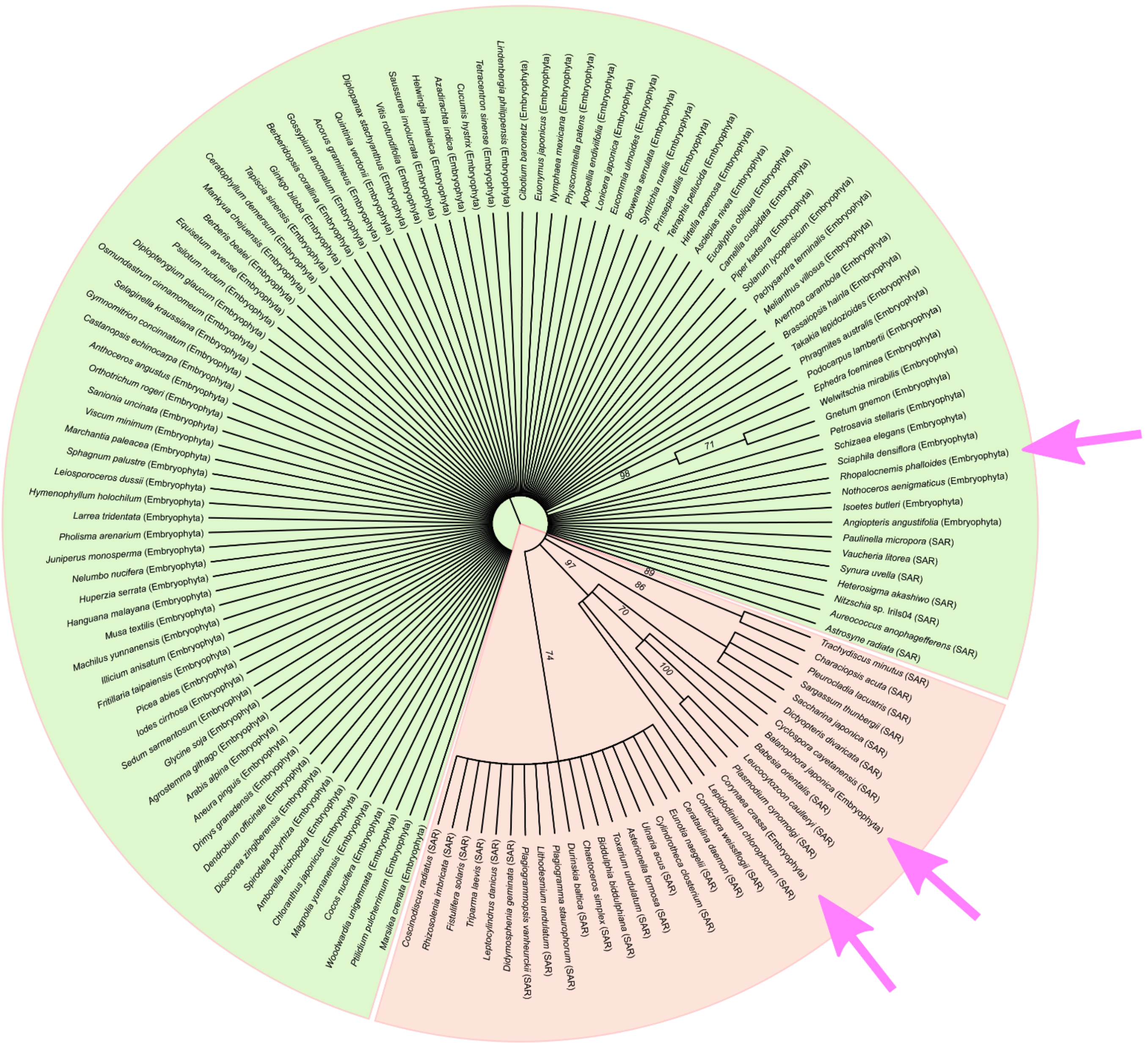

Figure S3. The radial cladogram of *rrn16* from Embryophyta and SAR, after the removal of alignment columns where *Rhopalocnemis phalloides* had adenines or thymines. Branches with boostrap support below 70 are collapsed. The placement of the three Balanophoraceae is indicated by pink arrows. *R. phalloides* is situated in a poorly resolved group with all Embryophyta (except the two other Balanophoraceae) and several representatives of SAR. This group is coloured green, while other groups containing only SAR, *Corynaea crassa*, and *Balanophora japonica* are coloured orange.

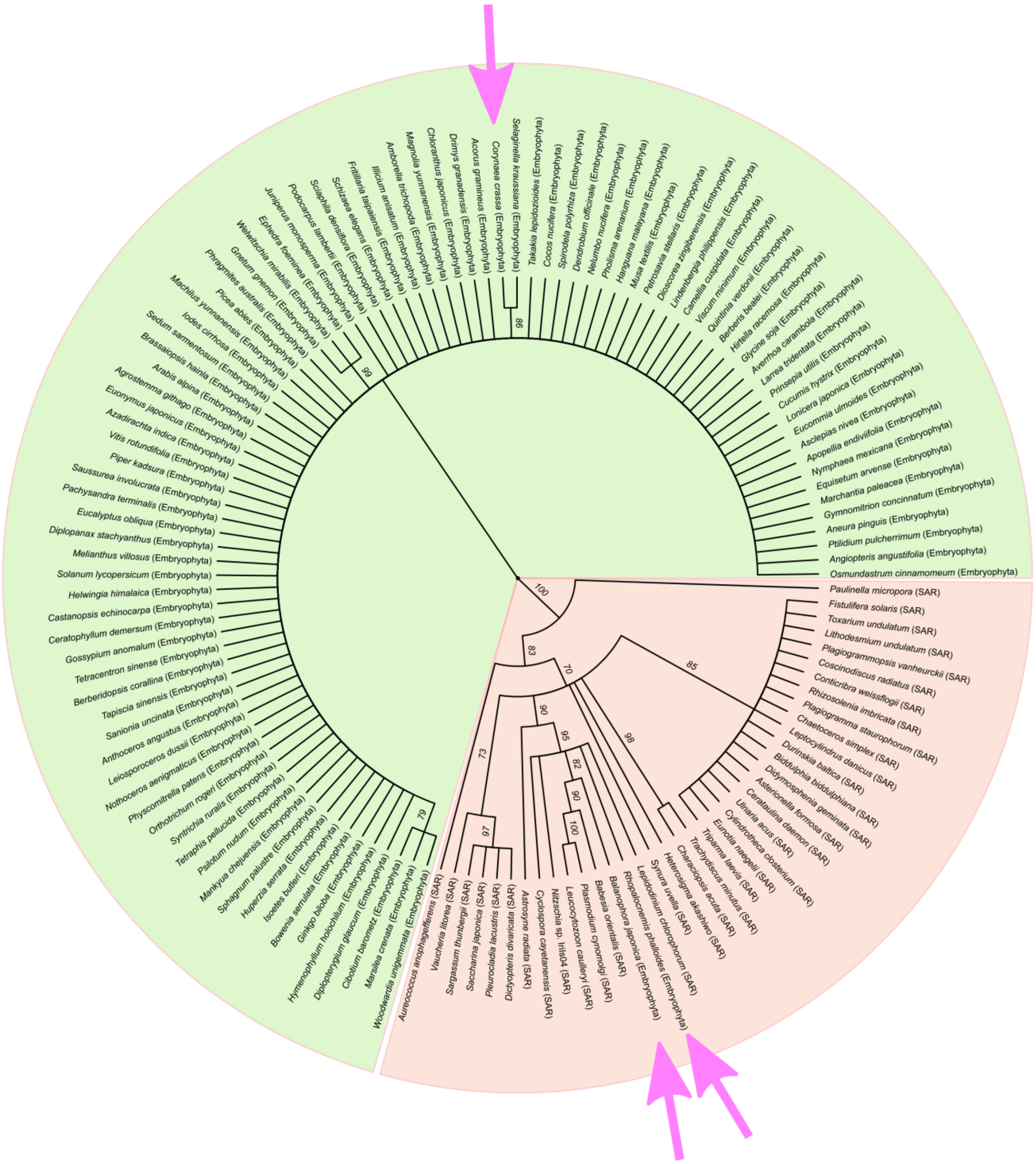

Figure S4. The radial cladogram of *rrn16* from Embryophyta and SAR, after the removal of alignment columns where *Corynaea crassa* had adenines or thymines. Branches with bootstrap support below 70 are collapsed. The placement of the three Balanophoraceae is indicated by pink arrows. *C. crassa* is situated in a poorly resolved group with all Embryophyta except the two other Balanophoraceae. This group is coloured green, while the group with SAR, *Rhopalocnemis phalloides*, and *Balanophora japonica* is coloured orange.

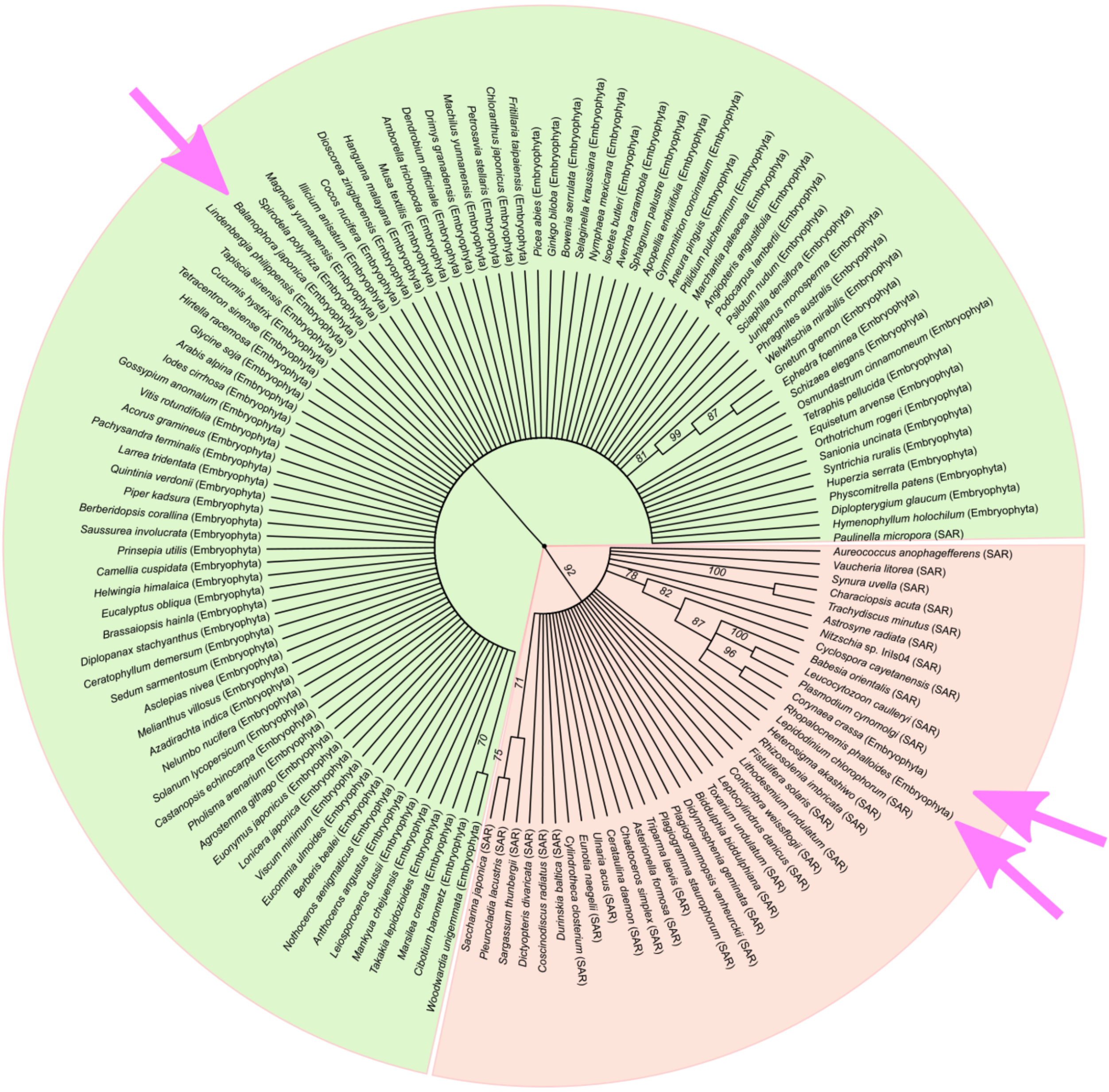

Figure S5. The radial cladogram of *rrn16* from Embryophyta and SAR, after the removal of alignment columns where *Balanophora japonica* had adenines or thymines. Branches with bootstrap support below 70 are collapsed. The placement of the three Balanophoraceae is indicated by pink arrows. *B. japonica* is situated in a poorly resolved group with all Embryophyta (except the two other Balanophoraceae) and *Paulinella micropora* (a representative of SAR). This group is coloured green, while the group with other representatives of SAR, *Rhopalocnemis phalloides*, and *Corynaea crassa* is coloured orange.

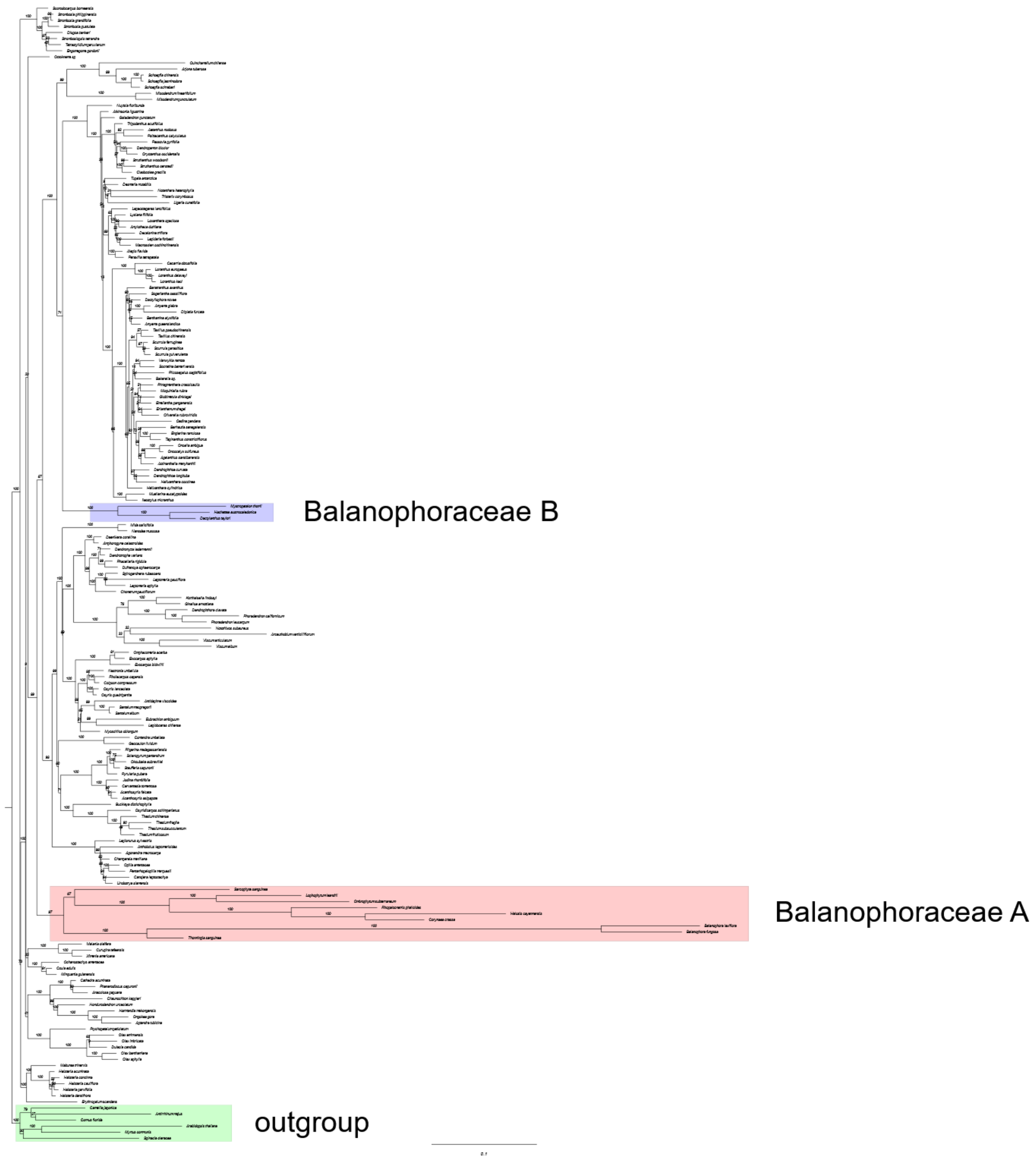

Figure S6. Phylogenetic tree of Santalales. The tree was built using a supermatrix of seven genes. The phylogenetic placement of *Rhopalocnemis phalloides* was determined using only two of its genes, namely the nuclear 18S rDNA and 26S rDNA. Numbers over branches denote bootstrap support values. *Antirrhinum majus*, *Cornus florida*, *Camellia japonica*, *Spinacia oleracea*, *Myrtus communis* and *Arabidopsis thaliana* are used as the outgroup. The scale bar represents the number of nucleotide substitutions per position.

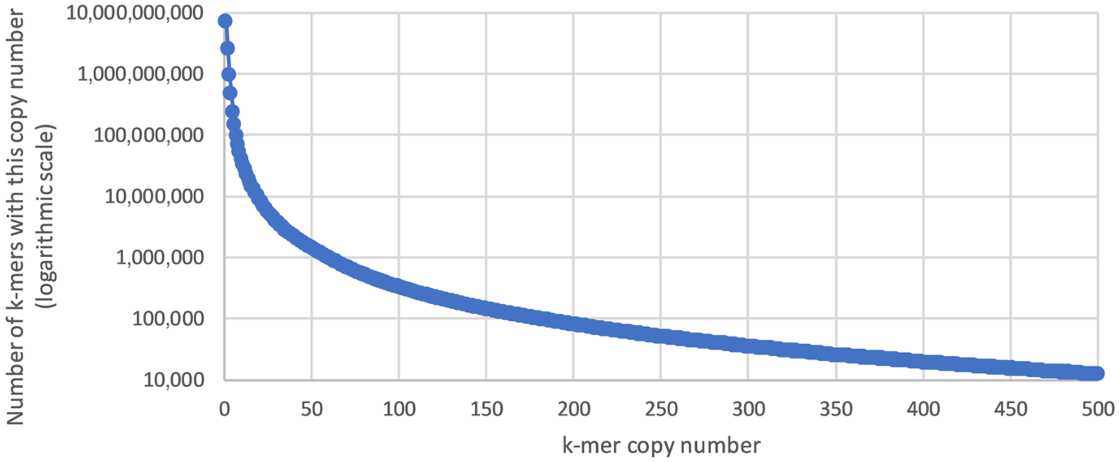

Figure S7. K-mer frequency spectrum of DNA-seq reads.
